# Patterns of Species Richness and Distribution of the Genus *Laelia* s.l. vs. *Laelia* s.s. (Laeliinae: Epidendroideae: Orchidaceae) in Mexico: Taxonomic Contribution and Conservation Implications

**DOI:** 10.3390/plants11202742

**Published:** 2022-10-17

**Authors:** Ma. Isabel Olivares-Juárez, Mireya Burgos-Hernández, Margarita Santiago-Alvarádo

**Affiliations:** 1 Departamento de Biología, Universidad Autónoma Metropolitana, Unidad Iztapalapa, Av. San Rafael Atlixco 186, Leyes de Reforma, 1ra sección, Iztapalapa, Mexico City 09340, Mexico; 2 Programa de Posgrado en Botánica, Colegio de Postgraduados, Km 36.5 carretera Mexico-Texcoco, Montecillo, Texcoco 56230, Mexico; 3 Laboratorio de Biología Comparada, Facultad de Estudios Superiores Zaragoza, Universidad Nacional Autónoma de México, Batalla 5 de mayo sn, Ejercito de Oriente, Iztapalapa, Mexico City 09230, Mexico

**Keywords:** conservation, neotropics, orchids, potential distribution models, taxonomy

## Abstract

*Laelia* is an endemic genus of the neotropical region, with the greatest richness occurring in Mexico. A recent phylogenetic study transferred some Mexican laelias to the genus *Schomburgkia*, which has generated debate. The aim of the present study was to analyze the patterns of species richness and endemism and the current and potential geographic distributions of the taxa of *Laelia* s.l., as well as the putative *Laelia* s.s., distributed in Mexico as part of an exploratory evaluation of the generic limits to sheds light on the taxonomic debate and generate baselines to guide conservation efforts. A database was generated with information from herbarium specimens and publications. The species richness was estimated by political division, biomes, and elevation. The endemism was analyzed by political division and using the weighted and corrected weighted endemism indices. Geographic data, climatic, and topographic variables were used to predict the distributions with the maximum entropy algorithm. The results supported the proposal to transfer some species to the genus *Schomburgkia*. Some areas of the Sierra Madre del Sur and Oriental should be included as priority areas in the conservation strategies of *Laelia*. This study highlights the importance of the taxonomy, distribution, and hotspots in diversity conservation.

## 1. Introduction

*Laelia* Lindl. *sensu lato* (s.l.), is a genus comprising 25 species of epiphytic orchids restricted to the neotropical region, with its main center of diversity being in Mexico, but with a greater affinity towards the mountains [1,2]. The species of the genus are characterized by having articulated leaves at the apex of the pseudobulb, which are coriaceous, fleshy, carinate, conduplicate, and subpetiolate, as well as by their large and showy flowers [1,3]. Due to their conspicuous inflorescence, laelias have great ornamental importance and have stood out for their ceremonial and horticultural use. They have also drawn attention for their uses as a raw material for the manufacture of adhesive, healing, binder, and culinary products [1,4]. In Mexico, *Laelia albida* Bateman ex Lindl., *Laelia anceps* Lindl., *Laelia autumnalis* (Lex.) Lindl., *Laelia furfuracea* Lindl., *Laelia gouldiana* Rchb. f., and *Laelia speciosa* (Kunth) Schltr. are traditionally used to decorate offerings and tombs, as well as in traditional medicine to treat hemorrhages, wounds, dysentery, inflammation, and fever [5,6].

Given the problems in the delimitation of the genus and species, to date there is no consensus on the number of species recognized in Mexico. Halbinger and Soto-Arenas [1] reviewed the taxonomy of Mexican laelias and recognized 11 species, two subspecies, and four hybrids, with nine taxa reported as endemic. Villaseñor [7], in his list of Mexican vascular plants, listed 12 species and a natural hybrid, with 10 being endemic. Solano et al. [8] estimated—based on a review of various studies—the presence of 13 species and four natural hybrids. One year later, Solano and other authors, in their updated catalog of taxonomic authorities of Orchidaceae of Mexico, reduced the number of natural hybrids in the national territory to three [9].

The genus *Laelia* has a long history of taxonomic changes, including the combination of *Schomburgkia* Lindl. with *Laelia*, which had already been proposed by Williams [10] and taken up by Van den Berg and Chase [11]. A recent phylogenetic study carried out by Peraza-Flores et al. [12], where 21 of the 25 accepted species [2] in the entire genus *Laelia* s.l. were evaluated, found two strongly supported clades, one corresponding to elements endemic to Mexico and another that includes the taxa that were originally circumscribed to the genus *Schomburgkia*, whose species have a wider distribution. After analyzing the results and re-evaluating the morphology, as well as the taxonomic delimitation, the authors recognize both genera as valid and independent. Additionally, they transferred *Laelia anceps*, *L. aurea* A.V.Navarro, *L. dawsonii* (J.Anderson) De B.Crawshay, *L. halbingeriana* Salazar and Soto Arenas, *L. rubescens* Lindl., *L. superbiens* Lindl., and *L.* × *oaxacana* Salazar and R.Jiménez to *Schomburgkia*, leaving only *L. albida*, *L. autumnalis*, *L. crawshayana* Rchb.f., *L. eyermaniana* Rchb.f., *L. furfuracea,* and *L. speciosa* within *Laelia sensu stricto* (s.s.). Based on their results, the authors also treated *L. gouldiana* as a hybrid under the name of × *Schombolaelia gouldiana* (Rchb.f.) Peraza and Carnevali, and indicated that the parents are *Schomburgkia anceps* × *Laelia autumnalis*. After this publication, several authors continued considering this taxon in the species range and to the genus *Laelia* in its broad delimitation [7,8,9,13].

Said changes will not only reduce the number of recognized species for Mexico from 13 to six but the distribution of the genus will be restricted to the Mexican territory. The above has profound implications for the management and conservation of *Laelia* species, highlighting the importance of generating different sources of evidence that contribute to resolving this taxonomic uncertainty and at the same time providing information that is useful for the implementation of adequate strategies for their conservation and management.

The knowledge about the current and potential distribution patterns of taxa and geographical areas with greater species richness provides essential information for understanding the processes that shape their diversity and generate basic information on conservation issues [14,15]. These models use associations between environmental variables and known localities to define the abiotic conditions within which populations and species can be maintained [16]. In this sense, potential distribution models constitute useful tools that have been used, among other things, to provide evidence of geographic isolation and identify priority areas [17]. Knowledge about the distribution and richness patterns of endangered and endemic species can be used to guide conservation efforts, especially in the delineation of new priority areas for conservation [18,19]. In addition, the application of potential distribution models in taxonomic boundary problems can provide significant evidence for this goal. The above points, on the basis that many environmental attributes, are critical for adaptive responses within and between species, and these adaptations are major forces driving speciation [20,21]. This proposal was initially discussed by Wiens and Graham [17], and precisely from their work various contributions have emerged in which these tools are included to evaluate aspects of taxonomic circumscription [22,23,24,25].

To date, ethnoecological [26], phylogenetic [12], genetic [27,28,29,30,31], horticultural [32,33,34] and morphological [35,36] aspects of the genus *Laelia* have been addressed. However, no studies have addressed the current or potential distribution and richness patterns of the genus on a national scale. To contribute to this knowledge and generate useful information to establish strategies for the conservation and management of the species, as well as to explore additional evidence that clarifies the correct circumscription of the group, the present study aimed to analyze the current and potential geographic distribution and richness patterns of the taxa of *Laelia* s.l., as well as *Laelia* s.s. and species proposed to be transferred to *Schomburgkia* distributed in Mexico, following the circumscription proposed by Peraza-Flores et al. [12].

## 2. Results

### 2.1. Database

A total of 1339 records of *Laelia* were obtained, of which 860 corresponding to duplicate data; cultivated species; and purchased, incomplete, or confusing information were excluded. The final database consisted of 479 records, of which 19% had to be georeferenced and 2.5% needed nomenclatural corrections since synonymy errors were found. Among the most notable cases were *Laelia crawshayana* Rchb.f., where 100% of its records were found under the name of *L. bancalarii* R.González and Hágsater, which is currently invalid. Likewise, the names *L. catarinensis* Rosillo and *Schomburgkia superbiens* (Lindl.) Rolfe were commonly found in the databases and are currently synonyms of *L. eyermaniana* Rchb.f. and *L. superbiens* Lindl. respectively. No species were classified in a risk category according to the IUCN Red List [37]. However, nine species (75%) are under a risk category (Table 1) according to the Mexican regulation NOM-059-SEMARNAT-2010 [38]. Among the latter, *L. gouldiana* stands out, as it is the only one in the category of ‘probably extinct in the wild’.

### 2.2. Patterns in Species Richness, Distribution, and Endemism: Laelia s.l. vs. Laelia s.s.

**Figure 1:**
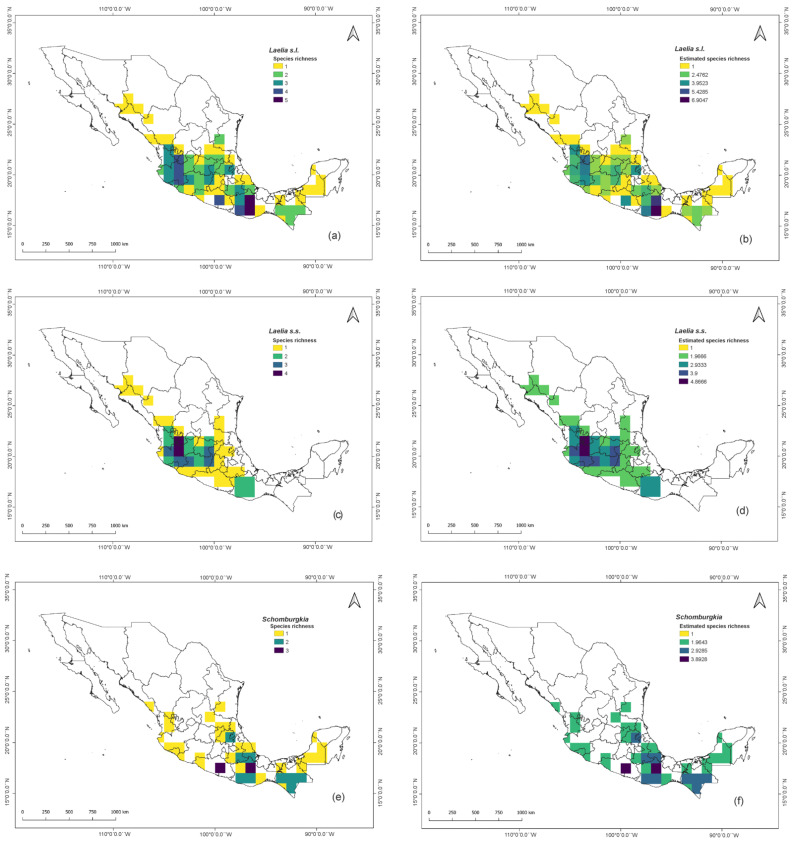
Species richness in Mexico for *Laelia* s.l.: (**a**) absolute richness; (**b**) estimated richness. Species richness in Mexico for *Laelia* s.s.: (**c**) absolute richness; (**d**) estimated richness. Species richness in Mexico for *Schomburkgia*: (**e**) absolute richness; (**f**) estimated richness.

In Mexico, there are 13 species and three hybrids of *Laelia* s.l., which are distributed in 24 of the 31 states of the country (Table 1). The states with the highest species richness were Oaxaca with seven species and three hybrids, Jalisco with six species, as well as Guerrero and Nayarit with five each (Table 1, Figure 1a). These data were corroborated by the results of the richness estimates of the non-parametric model Chao 1, since the cells that showed the highest estimated richness were located in these same areas (Figure 1b). Meanwhile, Campeche, Chihuahua, Morelos, Sonora, Tabasco, Veracruz, and Yucatán were only represented by one species, respectively.

**Figure 2:**
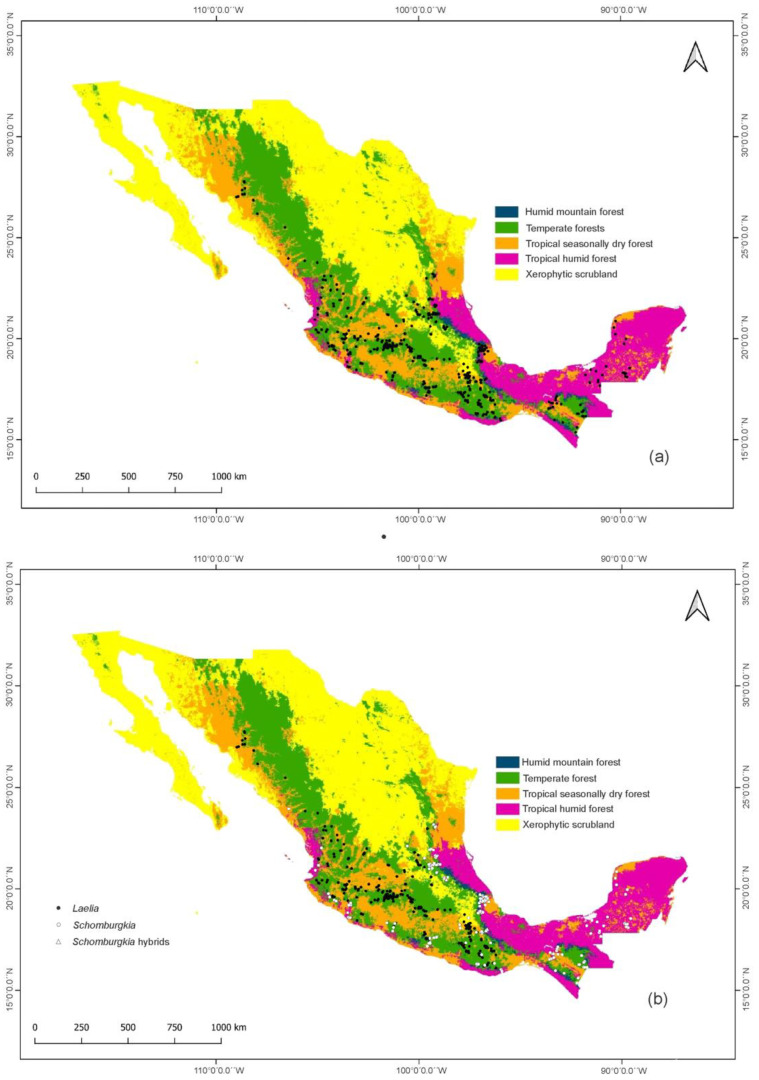
Distribution of (**a**) *Laelia* s.l., (**b**) *Laelia* s.s., and *Schomburgkia* in the five biomes of Mexico proposed by Villaseñor and Ortiz [39].

Of the five biomes reported for Mexico, the temperate forests stand out, where 11 species and two hybrids of *Laelia* s.l. are distributed, as well as the tropical seasonally dry forests with 10 species (Table 1). An important presence of the group is observed in forests of the central, western, and southwestern regions of the country. The humid mountain forests and the tropical humid forests constitute the biomes with the least representation of this genus, with only three species present. Since the group is essentially neotropical, it is not uncommon for it to have a smaller presence in the north of the country, especially in the xerophytic scrubland (Figure 2a).

**Figure 3:**
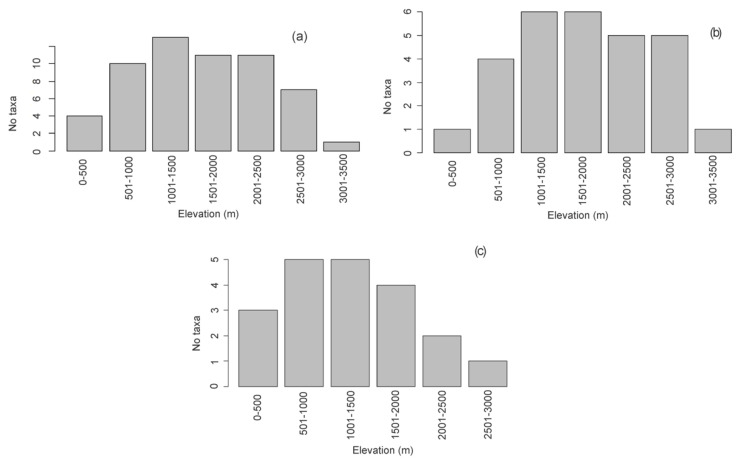
Number of taxa by elevation for (**a**) *Laelia* s.l., (**b**) *Laelia* s.s., and (**c**) *Schomburgkia*.

**Figure 4:**
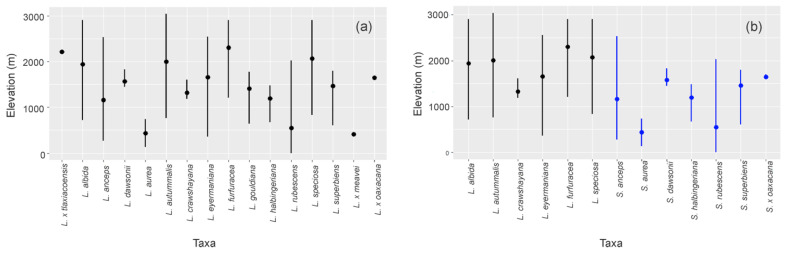
Elevation range by taxon for (**a**) *Laelia* s.l., (**b**) *Laelia* s.s., and *Schomburgkia*.

According to the elevation distribution analysis, *Laelia* s.l. is distributed in a range that goes from 0 to just over 3000 m, but with a greater affinity towards elevations between 501 and 2500 m. It stands out that 100% of the species grow at altitudes between 1000 and 1500 m (Figure 3a). According to the analysis by taxon (Figure 4a), *L. anceps* (272–2528 m) and *L. eyermaniana* (358–2850 m) had the widest altitudinal ranges. *Laelia autumnalis* is the only species that reaches altitudes above 3000 m, in contrast to *L. aurea*, whose upper elevational distribution range does not exceed 739 m, representing the most restricted elevational distribution range (135–739 m).

Of the species included in the genus, 77% (10 species) are endemic to Mexico (Table 1). Of these, *L. speciosa* was the one that registered the widest distribution in the country, located in 12 states, followed by *L. autumnalis* in 10 states. Jalisco (4), Nayarit (4), and Oaxaca (2) were the states with the highest number of endemic species, while *L. crawshayana* (Jalisco), *L. furfuracea* (Oaxaca), *L. gouldiana* (Hidalgo), and *L. halbingeriana* (Oaxaca) were not recorded beyond a state limit, for which reason they are considered microendemic in this study.

**Figure 5:**
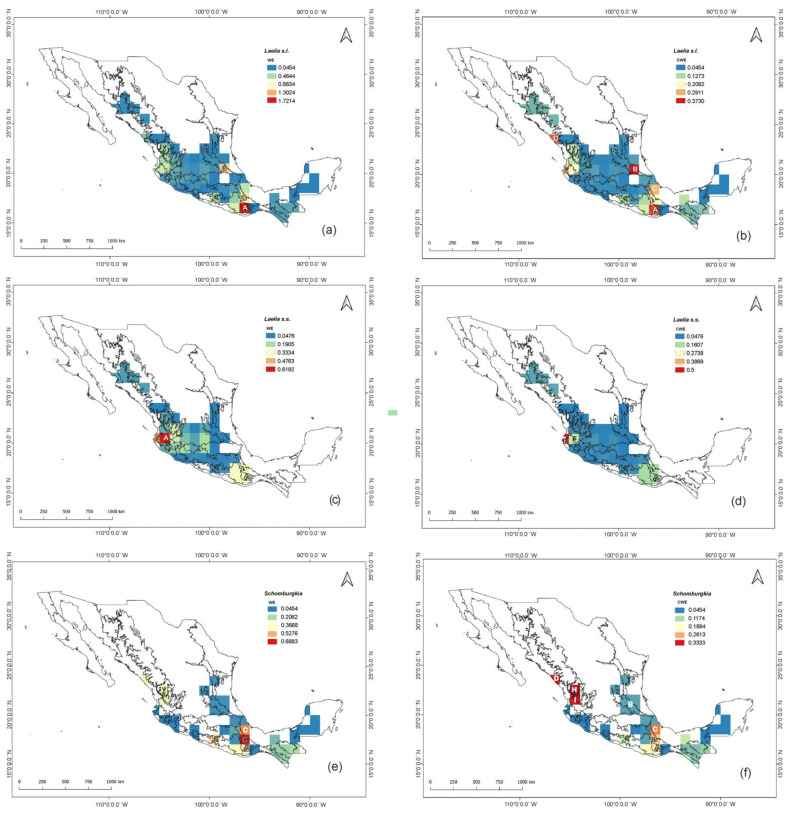
Weighted endemism index (WE) values for (**a**) *Laelia* s.l., (**c**) *Laelia* s.s., and (**e**) *Schomburgkia* and corrected weighted index (CWE) values for (**b**) *Laelia* s.l., (**d**) *Laelia* s.s., and (**f**) *Schomburgkia*.

The cells with the highest values of endemism discussed below are labeled with capital letters in Figure 5. The weighted endemism index (WE) (Figure 5a) showed two cells with the highest values of endemism. Cell A, with a value of 1.72, is located at the convergence of the Balsas Basin (BB) and Sierra Madre del Sur (SMS) biogeographic provinces in its largest area, and cell B, with an index of 1.12, is located in the Sierra Madre Oriental (SMOR). A third cell (cell C), also located between the BB and SMS provinces, shows a value of 0.88. This is similar to the absolute richness patterns reported (Figure 1a).

When endemism values are weighted to remove the influence of species richness (i.e., the corrected weighted index (CWE)), the results change slightly. Again, cells A and B recovered the highest values but were lower than those obtained with the WE index. Cell A, which previously had a value of WE = 1.72, now displays a value of CWE = 0.34, and cell B, which had a value of WE = 1.12, now shows a value of CWE = 0.37 (Figure 5b). Now, cell D located in the Pacific Lowlands (PL) ranks third in endemism, with an index of CWE = 0.28, followed by two cells, C (CWE = 0.27) and E (CWE = 0.20), located at the convergence of the PL and the SMS.

If we analyze the same patterns but now under the re-circumscription proposed by Peraza-Flores et al. [12], *Laelia* s.s. is represented by seven species and two hybrids (Table 1), while the other six species and the remaining hybrid would be circumscribed to the genus *Schomburgkia*. Considering this proposal, the patterns of distribution, richness, and endemism for both genera change markedly.

At the political division level, *Laelia* s.s. is distributed in 19 states (Table 1), five less than those reported for *Laelia* s.l.; these are Campeche, Chiapas, Tabasco, Veracruz, and Yucatan. The latter states then correspond to exclusive distribution areas of species transferred to *Schomburgkia*, which together with 11 more states, make up the entire distribution range of this last genus in the country. For their part, Aguascalientes, Chihuahua, Durango, State of Mexico, Guanajuato, Morelos, Sonora, and Zacatecas are states with an exclusive presence of *Laelia* s.s. The states with the greatest richness of this last genus are Jalisco (5), Guanajuato (3), Michoacan (3), and Nayarit (3) (Table 1, Figure 1c). Oaxaca (6), Chiapas (3), and Guerrero (3) stand out for being numerous in the species assigned to the genus *Schomburgkia* (Table 1, Figure 1e). Both patterns could be corroborated by the Chao 1 estimator, since these same areas were the ones that showed the highest estimated richness for the two genera (Figure 1d,f). It should be noted that *Laelia* s.s. does not have a presence in southeastern Mexico, contrary to what happens with *Schomburgkia*.

Regarding the distribution of *Laelia* s.s. by the type of biome (Figure 2b), a greater number of taxa are recorded as occurring in temperate forests (6), followed by tropical seasonally dry forests and xerophytic scrublands, with five species each. The absence of the genus in tropical humid forests and the presence of only one species (*L. albida*) distributed in humid mountain forests stand out. The species proposed as part of *Schomburgkia* show a greater affinity towards tropical seasonally dry forests and temperate forests, with five species each, followed by tropical humid forests with four species. On the other hand, the xerophytic scrublands are the least represented, with only *S. anceps* distributed in this biome. It is also possible to observe a distribution pattern with a greater affinity towards the lowlands of the Gulf and South Pacific slopes for this last genus. Meanwhile, *Laelia* s.s. shows its greater distribution towards the central-western and central-southern mountainous regions of the country.

Differences between the elevation ranges in which the species of both genera are distributed could be observed. Thus, *Laelia* s.s. develops between 358 and 3041 m a.s.l., with a greater affinity towards elevations between 1700 and 2500 m (Figure 3b). *Laelia crawshayana* was the species with the most restricted elevation distribution range within the genus, as it only grows between 1185 and 1607 m (Figure 4b). For their part, the species that integrate the genus *Schomburgkia* grow on average below 2000 m (Figure 3c), except for *S. anceps*, which has an upper altitude limit of 2528 m (Figure 4b). The species of this genus have a greater affinity towards elevations between 1000 and 1800 m, contrasting with the preferences of *Laelia* s.s. *Schomburgkia dawsonii* was the species with the most restricted elevational range, growing only between 1447 and 1835 m (Figure 4b).

The endemism patterns also change dramatically when the proposed re-circumscription is considered. Thus, 100% of the species of *Laelia* s.s. are endemic, with the largest number occurring in Jalisco (4), Guanajuato, Michoacan, and Nayarit (3 each), with one being microendemic in Jalisco and another in Oaxaca. The largest number of endemic species assigned to *Schomburkgia* was found in Oaxaca (3), with two of them being microendemics. The WE index again showed two cells with the highest values of endemism, but these did not correspond to those detected for *Laelia* s.l. Cell F showed the highest index (WE = 0.61), followed by cell E (WE = 0.5), both located at the convergence of the PL and the SMS (Figure 5c). The same cells were recovered by the CWE index (Figure 5d). However, cell E was the one that obtained the highest index (CWE = 0.5), while cell F obtained a value of CWE = 0.20. Both cases showed slightly lower values than those obtained with the WE index. In the case of the species assigned to *Schomburgkia*, the maximum value of the WE index was 0.68 in cell G, located in the SMS, followed by cell C (WE = 0.57) located in the SMOR and cell J (WE = 0.45) in the SMS (Figure 5e), as well as cell A (WE = 0.40) in the SMS. In contrast, the CWE index (Figure 5f) showed cells D, H, and I as having the highest values (CWE = 0.33 each), located in the SMOC and PL. They were followed by cells C (CWE = 0.28) and G (CWE = 0.23) in the SMS and BB. The rest of the cells maintained values below 0.20.

### 2.3. Potential Distribution Models

The distribution of 85% of the taxa was modeled, except for *L. aurea*, *L. dawsonii*, *L.* × *meavei*, and *L.* × *tlaxiacoensis*, which had very few records for this analysis. Four to 16 uncorrelated variables were selected to model the distribution of the different taxa and are listed in Supplementary Tables S1 and S2.

**Figure 6:**
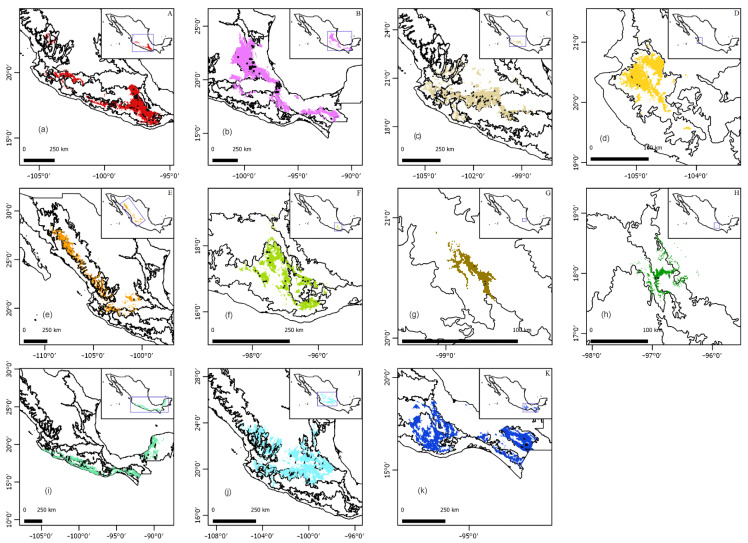
Potential distribution areas of (**a**) *Laelia albida*, (**b**) *Laelia anceps, (**c**) *Laelia autumnalis*, (**d**) *Laelia crawshayana*, (**e**) *Laelia eyermaniana*, (**f**) *Laelia furfuracea*, (**g**) ^×^*Laelia gouldiana*, (**h**) *Laelia halbingeriana, (**i**) *Laelia rubescens, (**j**) *Laelia speciosa*, and (**k**) *Laelia superbiens. *Taxa proposed to be transferred to *Schomburgkia* and ^×^taxon proposed in a new combination ×*Schombolaelia gouldeana* by Peraza-Flores et al. [12].

The models presented AUC values similar to 0.8 and higher than 0.9, so the performance was good, while in others it was excellent according to Peterson et al. [40]. The partial ROC tests yielded values greater than 1 (Table 2), classifying it as a good model following the proposal by Peterson et al. [41]. According to the analyzed models (Figure 6), the distribution range of *Laelia* s.l. is extended to other biogeographic provinces (Table 3), and except *L. gouldiana*, the potential distributions of the species overlap in certain areas of the SMS. The distribution of *Laelia* s.s. does not cover the Chiapas Highlands (CH), Tamaulipas (TAM), PL, Veracruzan (VE), or the Yucatan Peninsula (YP)’s biogeographic provinces. One of the results worth highlighting is that the potential distribution of the species assigned to *Schomburgkia*, except for *S. anceps* and *S. rubescens*, is limited by the Trans-Mexican Volcanic Belt province (TMVB), since none of them is present in this province.

**Figure 7:**
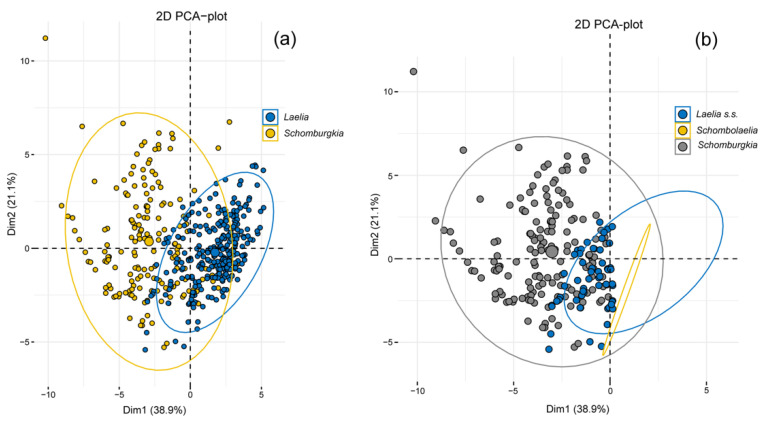
The principal component analysis (PCA) of the bioclimatic and topographical variables of the species of (**a**) *Laelia* s.s. and *Schomburgkia* and of (**b**) *Laelia* s.s., *Schomburgkia,* and ×*Schombolaelia*.

**Figure 8:**
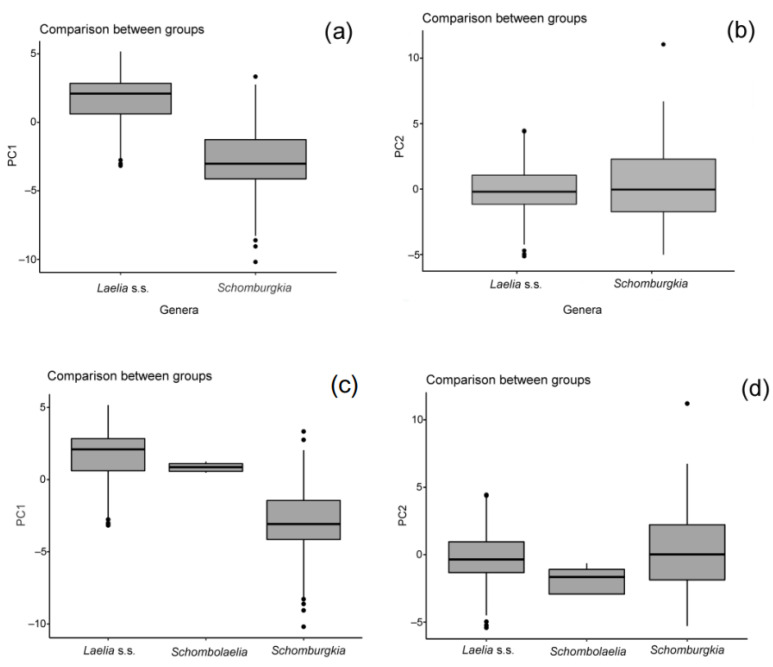
Comparison of means between genera by principal component by Tukey’s method, applying the Bonferroni adjustment between *Laelia* s.s. and *Schomburgkia*: (**a**) component 1; (**b**) component 2. Comparison of means between genera by principal component by Tukey’s method, applying the Bonferroni adjustment between *Laelia* s.s., *Schombolaelia*, and *Schomburgkia*: (**c**) component 1; (**d**) component 2.

According to the PCA carried out to examine the grouping patterns based on abiotic variables of the putative two genera, this showed that cumulatively the first two components explained 60% of the variance contained (Figure 7a). The first component was associated with the elevation and mean annual temperature (bio1), while the second component was mainly associated with the annual precipitation (bio12) and annual potential evapotranspiration (annualPET) (Table S3, Figure S1). The results of the ANOVA (*p* = < 2 × 10^−16^) and ANOSIM (R = 0.5993, *p* = 0.0001) showed significant differences between the climatic variables for both putative genera (Figure 8a,b).

When ×*Schombolaelia gouldeana* was included in the PCA (Figure 7b), it was grouped within the convergence space between *Laelia* s.s. and *Schomburgkia*. According to the ANOVA (*p* = < 2 × 10^−16^) and ANOSIM (R = 0.6062, *p* = 0.0001), significant differences were detected. These differences were identified as occurring between ×*Schombolaelia* and *Schomburgkia*, as well as between *Laelia* s.s. and *Schomburgkia* in PC1, and only between *Laelia* s.s. and *Schomburgkia* in PC2 (Table 4, Figure 8c,d).

## 3. Discussion

### 3.1. Patterns in Species Richness, Distribution, and Endemism: Laelia s.l. vs. Laelia s.s.

A total of 479 unequivocal records of the genus *Laelia* s.l. were analyzed and corresponded to 13 species and two hybrids inhabiting Mexico. One more hybrid, *L.* × *oaxacana*, was not included in the analysis, since the coordinates of its records were not available. This cipher confirms the number of species listed by Villaseñor [7] and coincides with the number of hybrids reported by Solano et al. [9] for this group.

Although the genus is widely distributed throughout the Mexican territory, it has a greater affinity towards temperate forests, mainly in the western and southwestern regions of the country, which register the greatest diversity. Consequently, it is not uncommon to find low species richness in the lowlands of the southeast of the country, specifically in the Yucatan Peninsula, given its abiotic characteristics and dominant humid tropical forest [42,43]. Similarly, the scarce presence of the group in the north of the country, in the xerophytic scrubland biome, is not surprising, since an arid climate typical of the nearctic region dominates here, making it unfavorable for the establishment of a large number of groups [44] and orchid endemism [42], especially from the subtribe Laeliinae, whose species are strictly neotropical [45].

According to our results, Oaxaca is the state with the largest number of taxa and estimated richness, representing 60% of the diversity of laelias. Only seven species and one hybrid had been reported in this state: *L. albida*, *L. anceps*, *L. autumnalis*, *L furfuracea*, *L. rubescens*, *L. speciosa*, *L. superbiens,* and *L*. × *oaxacana* [7]. After the revision carried out in the present work, we added two more hybrids to this list, *L*. × *meavei*, and *L*. × *tlaxiacoensis*, increasing the richness of laelias in that state from seven to nine taxa. This is not surprising, since Oaxaca is the state with the greatest floristic diversity [7,39], in addition having the largest number of Orchidaceae species in Mexico [46,47].

Regarding endemism, Halbinger and Soto-Arenas [1], Villaseñor [7], and Solano et al. [9] have reported different numbers for Mexico, with nine, 10, and 11, respectively. Our data differ from the last author, since we do not consider *L. anceps* as endemic, because it is also distributed in countries such as Guatemala and Honduras [1,48]. However, we add one more to those mentioned by Halbinger and Soto-Arenas [1], *L. halbingeriana*, reported by Salazar et al. [49]. According to our review, about 80% of the species are endemic to the Mexican territory, and although Jalisco, Nayarit, and Oaxaca stand out at the level of political division, it is essential to know the points of endemism at the biogeographic level, since the distributions of the species obey less artificial factors [50]. Under this context, the biogeographical provinces SMS and BB stand out. The first is dominated by temperate forests with altitudes above 1000 m. Meanwhile, the second is neotropical and is located below 2000 m in altitude [51], in addition to having a close relationship with the SMS province [52]. Considering that the species of the genus show a greater affinity to temperate vegetation from humid to sub-humid climates and at elevations ranging from 1000 to 1500 m, it is not surprising that these provinces represent the main points of endemism for laelias. Something similar occurs with the province of PL and the SMOR in its convergence with the Veracruzana province, which is recovered as another important point of endemism. Particularly, this point of convergence is characterized by being very humid, dominating temperate and tropical forests [53], providing the necessary conditions for the establishment of the study group. Likewise, the SMOR hosts a high biological diversity, since not only do different biomes converge, but it also comprises a set of mountain ranges where pine and oak forests predominate, with altitudes above 1500 m [52,53], favoring the presence of this group of orchids. Several studies have documented a high richness and endemicity in these provinces. For example, Salinas-Rodríguez [54] reported 207 species and 68 genera of orchids occurring in the SMOR. Recently, Aragón-Parada et al. [55] reported the presence of 123 endemic species of Orchidaceae within the SMS, five of them from the genus *Laelia*. According to Morrone [52], these provinces are threatened by tourism, overgrazing, urbanization pressures, wildlife exploitation, and agricultural and livestock activities [56], which together can drive the most vulnerable taxa of the group to extinction.

Although 75% of the *Laelia* species in Mexico are classified as at risk according to NOM-059-SEMARNAT-2010 [38], there are still four species without any type of protection in the country. Furthermore, at the international level, there is no risk categorization for *Laelia*. Therefore, it is necessary to generate more information from different disciplines that allow Mexican laelias to be included in the red list IUCN. *Laelia gouldiana* is the only species listed as probably extinct. According to Menchaca and Moreno [57] and Bertolini et al. [58], this species is probably extinct in the natural environment as a result of over-collection and the modification of its habitat.

When we consider the re-circumscription of the taxa of *Laelia* s.l. proposed by Peraza-Flores et al. [12] in *Laelia* s.s. and *Schomburgkia*, important changes were detected in the configuration of their patterns of distribution, richness, and endemism. *Laelia* s.s. has an affinity for temperate forests and mountainous areas in the west and center of the country, showing differences from *Schomburkgia*, which shows a preference for seasonally dry tropical forests and lowland moist forests on the Gulf and Pacific slopes. This supports the assertion of Halbinger and Soto-Arenas [1], who mention that Mexican laelias are mostly mountain dwellers. This is important if we consider that the temperate forest is one of the largest biomes in the country; it is considered a critical and important ecosystem due to its high diversity and high level of endemism [59,60,61,62].

### 3.2. Potential Distribution Models

Unlike previous studies [8,49,63,64], in the present study, most species of the genus *Laelia* s.l. in Mexico were modeled. The potential distribution of each species allowed the limits of their distribution to be inferred, and unlike an approximation made under any approach or method of areography, this is defined by the coincidence in environmental conditions.

Most of the taxa of *Laelia* s.l., *Laelia* s.s., and *Schomburgkia* are concentrated in the SMS and the TMVB, with both provinces belonging to the Mexican Transition Zone (MTZ). The MTZ is a varied and complex area where nearctic and neotropical biotas overlap [65,66,67]. The SMS has been characterized by its complex geological and paleoclimatic history that has favored the presence of high biological diversity, and it is a clear example of the tendency of the Mexican territory to present a greater diversity of species in the south [68,69]. Salazar et al. [49] mention that *L. halbingeriana* and *L.* × *oaxacana* are distributed in the SMOR. However, Aragón-Parada et al. [55] recorded both together with *L. crawshayana*, *L. furfuracea,* and *Laelia* × *meavei* in their catalog of endemic vascular plants of the SMS, which partially coincides with our results. Some species such as *L. eyermaniana* and *L. speciosa* have extended their distribution to this province, contrary to what was reported by Halbinger and Soto-Arenas [1]. Consequently, we propose that this geographical area and the SMOR (of Puebla to Tamaulipas in their convergence with Nuevo Leon) be included, with higher priority, in the conservation strategies for *Laelia* species as micro-reserves. This is especially important when hotspots and endemism areas are located throughout heterogeneous environments such as the SMS and SMOR. At the same time, other species would be protected, as proposed by Salinas-Rodríguez et al. [70] and Aragón-Parada et al. [55].

According to our results, the Isthmus of Tehuantepec seems to constitute an important geographical barrier for the genus *Laelia* s.s., which had already been suggested by Halbinger and Soto-Arenas [1]. In addition, this last group shows a greater affinity towards the center and west of the country. In contrast, the species of laelias proposed to be transferred to *Schomburgkia* are distributed throughout the Isthmus, particularly *S. anceps*, *S. rubescens*, and *S. superbiens*. Large changes in the distribution patterns of many groups have been reported in this area [65,71], and it has been showing the role of the Isthmus of Tehuantepec as a biogeographical barrier in plant dispersal [72].

The results obtained from the climatic variables showed that the genera are statistically different in their environmental space, which together with the data on the distribution support the proposal by Peraza-Flores et al. [12] to transfer some of the *Laelia* species distributed in Mexico to the genus *Schombugkia*. Taking into account the previous morphological and phylogenetic evidence [12], as well as the geographical and environmental evidence generated in this study, it is argued that the genus *Laelia* s.s. is endemic to Mexico, which has important implications for conservation and management. Therefore, the conservation of *Laelia* s.s. should be prioritized if it is proven to be an endemic genus to Mexico.

In addition, our statistical analyses also support the new combination for *Laelia gouldiana* as ×*Schombolaelia gouldiana* according to Peraza-Flores et al. [12], which has an important impact on conservation issues. As mentioned above, this taxon is considered probably extinct, and various conservation efforts have been directed towards it [57]. Soto-Arenas and Solano-Gómez [73] had already reported that this taxon had been described from specimens without precise data of origin and that the only known specimens came from cultivated plants, located in the Metztitlán ravine in Hidalgo, a place where to date no specimens have been located again. Consequently, the conservation status of *L. gouldiana* must be analyzed in depth, and we must establish whether or not it is a hybrid, as supported here. Accurately identifying plants is a huge challenge, even more so in organisms that we are interested in protecting. Hence, taxonomic changes can affect the conservation status of organisms, as emphasized in several studies [74,75,76,77,78].

## 4. Conclusions

Although taxonomy and conservation are not the same, they do go hand in hand [74,75,76,77,78]; therefore, the proposed taxonomic changes imply, at the same time, a reassessment of the conservation status of the genus and encourage further progress in the group. Considering the current threat to the ecosystems present in the neotropics [79,80], as well as anthropogenic climate change, *Laelia* populations are highly vulnerable, and studies should continue to be carried out to allow adequate measures to be taken for their correct management, as well as for the establishment of efficient strategies for their conservation. In this sense, the hotspots detected and the distribution and endemism patterns identified in this study should be used as references to establish and focus efforts on conservation in situ. In particular, the patterns described are important to define priority sites and areas for the conservation of these species, since among the attributes associated with the narrow geographical distribution are a local and global rarity, isolation, as well as functional diversity [14,81,82]. Our work provides information for both the taxonomic study and conservation management of *Laelia* and highlights the integrated importance of taxonomy, distribution patterns, and richness hotspots in diversity conservation.

## 5. Materials and Methods

### 5.1. Database

A preliminary list of species was compiled from an exhaustive review of the floristic–taxonomic literature on the genus *Laelia* in Mexico [1,7,8,12]. Based on the consultation of digital databases such as the GBIF (https://www.gbif.org/ (accessed on 10 February 2022)), National Herbarium of Mexico (MEXU) (http://www.ib.unam.mx/botanica/herbario/ (accessed on 1 January 2022)), Network of Herbariums of Northwest Mexico (https://herbanwmex.net/portal/ (accessed on 15 January 2022)), SNIB-MX (http://www.snib.mx/ (accessed on 8 March 2022)), and Tropicos (www.tropicos.org (accessed on 10 March 2022)), as well as databases of herbaria such as CHAPA, CIB, CIIDIR, CITRO, CORU, EBUM, EAP, GBH, GUADA, HNMN, HUUA, LAGU, PMA, QMEX, SLPM, TEFH, UADY, UAS, WLM, XAL, and ZEA [83], the records of the species were obtained. A database was generated in Microsoft^®^ Excel v15.0. with the information collected.

The names of the species and their authors were verified on the web pages The International Plant Names Index (www.ipni.org (accessed on 12 April 2022)) and Plants of the World Online (http://powo.science.kew.org/ (accessed on 12 April 2022)). The risk statuses were consulted from the IUCN Red List [37] and NOM-059-SEMARNAT-2010 [38]. The geographic coordinates were corrected and inferred when necessary in the Google^®^ Earth Pro v7.3 (Google, Kansas, MO, USA) using the description of the collection location.

Homotypic data cleaning was carried out on the information collected with the mmqgis plugin in QGIS v3.10.9 [84,85]. To eliminate atypical records (outliers), the database records were intercepted with the 19 bioclimatic variables from WorldClim [86], one bioclimatic variable from ENVIREM [87], and three topographic variables from EarthEnv [88] (Table S1). Dendrograms were built showing the similarity relationships between records to discriminate between them using R v4.2.1 [89]. Finally, dense log clouds with a distance of 1000 m were reduced with the spThin package [90] in RStudio v554 [91].

### 5.2. Patterns in Species Richness, Distribution, and Endemism: Laelia s.l. vs. Laelia s.s.

The patterns of richness, distribution, and endemism for the species of *Laelia* s.s. were analyzed following the proposed circumscription of the genus according to Peraza-Flores et al. [12]. Meanwhile, the patterns of *Laelia* s.l. were analyzed following Van den Berg and Chase [11] and Solano et al. [9].

The species richness for each set of taxa was quantified and analyzed by political division. Subsequently, to recognize areas with high numbers of absolute species, richness cells of 1° latitude × 1° longitude were used in Biodiverse v3.1. [92,93] using a political division map on a scale of 1:250,000 [94]. In addition, the estimation of richness was carried out using the Chao 1 non-parametric model, which is based on abundances [95] and implemented in the same program. To describe the known distribution, the biome classification of Villaseñor and Ortiz [39] was used. The endemism was first assessed using political limits. When the distribution of a taxon was restricted to the Mexican territory, it was considered an element endemic to the country, and it was considered microendemic when no distribution record was found outside a state limit. A second assessment was made by estimating the weighted endemism index (WE) and the corrected weighted index (CWE) proposed by Crisp et al. [96] and Linder [97], both implemented in Biodiverse v3.0 software. Taking into account that endemisms are relevant to the knowledge and understanding of the evolutionary history, the cells with the highest level of endemism were overlapped for analysis, with a map of the biogeographical provinces of Mexico [52,98], using a cell size of 1° latitude × 1° longitude. In parallel and from the elevation data of the collections of the herbarium specimens, the altitudinal distribution of the species was analyzed. The presence points were categorized into 500 m classes and the number of taxa distributed in each class was plotted, as well as the elevation range for each taxon, with the support of RStudio v554 [91].

### 5.3. Potential Distribution Models

Potential distribution models were built only for those species with sufficient records, using the maximum entropy algorithm in MaxEnt v3.4.4 [99]. The delimitation of area *M* was defined based on the Hydrological Sub-Basins of Mexico [100] and America [101]. The latter was used to delimit the mobility space of the species *Laelia anceps*, *L. rubescens*, and *L. superbiens*, since they reach their distribution range outside of Mexico.

A first potential distribution training model was generated for each taxon, in which the following parameters were established: 75% of the points were used for model training and 25% to validate it with a bootstrap analysis with 100 repetitions and a convergence threshold of 0.00001. To avoid artificial extrapolations in the extreme values of the environment variables, the extrapolate and do clamping options were disabled [102]. From the jackknife test calculated in MaxEnt v3.4.4 and an r-Pearson correlation analysis performed in Rstudio v554, the most important variables were selected and used to make the final model for each species.

The models were evaluated with accuracy tests. The area under the curve (AUC) test was used, which is a measure of the receiver operating characteristic (ROC) analysis. According to Peterson et al. [40], models with AUC values between 0.7 and 0.9 are considered good and those with values above 0.9 are considered excellent. However, the usefulness of ROC analyses has been questioned, as they do not consider absences and do not give equal weighting to errors of omission and commission [41,103]. Therefore, the final models were validated through a partial ROC analysis in the NicheToolBox platform [104], which allows for differentiation between the AUC of the model prediction and the random AUC. Finally, we converted the potential distribution models from continuous to binary in order to locate potential presence areas. For this, the model was reclassified in Qgis, taking the minimum training presence threshold value from the MaxEnt results. The potential distribution of the modeled species was characterized according to the biogeographical provinces used by Morrone et al. [98].

In order to explore differences in the environmental conditions in which the two putative genera inhabit, a principal component analysis (PCA) was carried out, taking into account the values of the 23 bioclimatic and topographic variables associated with each of the records of the different species. To determine the existence of significant differences between groups, the values of the most explanatory variables from the PCA were compared using an analysis of variance (ANOVA). To explore whether the abiotic variables contribute to clarifying the taxonomic status of *L. gouldina*, a second PCA was carried out, integrating this species as a third group under the name of ×*Schombolaelia gouldiana*. In order to find out the differences between the three groups, a Tukey’s comparison of means was carried out. For both cases, a non-parametric post hoc analysis of similarities (ANOSIM) based on Euclidean distances was performed using all available variables and by applying the Bonferroni probability value (*p*) correction. All statistical analyses were carried out in RStudio v554.

## Figures and Tables

**Table 1:** Distribution of the species of *Laelia* s.l. in Mexico by political division, by biomes following the classification by Villaseñor and Ortiz [39], and by risk category according to the NOM-059-SEMARNAT-2010 [38].

Species	States	Biomes	Risk Category
* *Laelia albida* Bateman ex Lindl.	Guerrero, Jalisco, Nayarit, Oaxaca, Puebla	HMF, TF, TSDF, XS	-
^S^ *Laelia anceps* Lindl.	Chiapas, Guerrero, Hidalgo, San Luis Potosi, Oaxaca, Puebla, Querétaro, Tamaulipas, Veracruz	HMF, TF, TSDF, THF, XS	-
*^S^ *Laelia aurea* A.V.Navarro	Nayarit, Sinaloa	BTES, THF	T
* *Laelia autumnalis* (Lex.) Lindl.	Aguascalientes, Guerrero, Guanajuato, Jalisco, Mexico, Michoacan, Morelos, Nayarit, Puebla, Zacatecas	TF, TSDF	SP
* *Laelia crawshayana* Rchb. f.	Jalisco	TF	E
*^S^ *Laelia dawsonii* (J.Anderson) Crawshay	Guerrero, Oaxaca	TF	E
* *Laelia eyermaniana* Rchb. f.	Chihuahua, Durango, Guanajuato, Jalisco, Michoacan, Sinaloa, Sonora	TF, TSDF, XS	-
* *Laelia furfuracea* Lindl.	Oaxaca	TF, TSDF, XS	SP
* *Laelia gouldiana* Rchb. f.	Hidalgo	XS	PE
*^S^ *Laelia halbingeriana* Salazar and Soto Arenas	Oaxaca	TF, TSDF	SP
^S^*Laelia rubescens* Lindl.	Campeche, Chiapas, Guerrero, Jalisco, Michoacan, Nayarit, Oaxaca, Puebla, Tabasco, Yucatán	TF, TSDF, THF	-
* *Laelia speciosa* (Kunth) Schltr.	Aguascalientes, Durango, Guanajuato, Hidalgo, Jalisco, México, Michoacan, Nayarit, Querétaro, San Luis Potosi, Tamaulipas, Zacatecas	TF, TSDF, XS	SP
^S^*Laelia superbiens* Lindl.	Chiapas, Oaxaca	HMF, TF, TSDF, THF	T
*Laelia* × *meavei* Cetzal and E.A.Pérez-García	Oaxaca	TF	-
^S^*Laelia* × *oaxacana* Salazar and R.Jimenéz	Oaxaca	-	-
*Laelia* × *tlaxiacoensis* Solano and Cruz-García	Oaxaca	TF	-

* Endemic to Mexico. ^S^ Taxon transfer to *Schomburgkia* by Peraza-Flores et al. [12]. Abbreviations: HMF (Humid Mountain Forest); TF (Temperate Forest); TSDF (Tropical Seasonally Dry Forest); THF (Tropical Humid Forest); XS (Xerophytic scrubland); T (Threatened); PE (Probably Extinct in the Wild); E (Endangered); SP (Subject to Special Protection).

**Table 2:** AUC values from each potential distribution model and the partial ROC analysis.

Specie	AUC Values		Partial ROC Values		
	First Model	Second Model	AUC Ratio	AUC at 0.05	AUC at 0.5
*Laelia albida*	0.906	0.846	1.71512	0.8573766	0.4998921
*Laelia anceps*	0.939	0.877	1.772603	0.8862974	0.4999978
*Laelia autumnalis*	0.911	0.882	1.800182	0.9000443	0.4999739
*Laelia crawshayana*	0.950	0.896	1.904308	0.9521538	0.5
*Laelia eyermaniana*	0.969	0.937	1.851861	0.9259304	0.5
*Laelia furfuracea*	0.949	0.923	1.796921	0.8984603	0.5
*Laelia gouldiana*	0.985	0.977	1.978512	0.989256	0.5
*Laelia halbingeriana*	0.981	0.957	1.939727	0.9698633	0.5
*Laelia rubescens*	0.920	0.871	1.728172	0.8640831	0.4999983
*Laelia speciosa*	0.911	0.858	1.680408	0.8401278	0.4999539
*Laelia superbiens*	0.967	0.926	1.841885	0.9209425	0.5

**Table 3:** Current and potential distribution of *Laelia* s.l. by biogeographical provinces.

Taxa	Biogeographical Provinces	
	Current Distribution	Potencial Distribution
*Laelia albida*	BB, TMVB, SMOC, SMS, PL	* CD
*Laelia anceps*	CH, BB, TMVB, SMOR, SMS, PL, VE	* CD, * TAM
*Laelia aurea*	TMVB, PL	-
*Laelia autumnalis*	BB, CD, TMVB, SMOC, SMS	* SMOR, * PL
*Laelia crawshayana*	SMS	* TMVB, * PL
*Laelia dawsonii*	BB, SMS	-
*Laelia eyermaniana*	CD, TMVB, SMOC, PL	* SMS, * SMOR
*Laelia furfuracea*	BB, SMS, PL	* TMVB
*Laelia gouldiana*	SMOR	* TMVB
*Laelia halbingeriana*	BB, SMS	* VE
*Laelia* × *meavei*	SMS	-
*Laelia* × *oaxacana*	SMS	-
*Laelia rubescens*	CH, BB, YP, SMS, PL, VE	* TMVB
*Laelia speciosa*	CD, TMVB, SMOC, SMOR, PL	* BB, * SMS, * VE
*Laelia superbiens*	CH, SMS, VE	* BB, * PL
*Laelia* × *tlaxiacoensis*	SMS	-

Note: * New biogeographic provinces in which the potential distribution was recorded. Abbreviations: CH (Chiapas Highlands province); BB (Balsas Basin province); CD (Chihuahuan Desert province); TMVB (Trans-Mexican Volcanic Belt province); YP (Yucatan Peninsula province); SMOC (Sierra Madre Occidental province); SMOR (Sierra Madre Oriental province); SMS (Sierra Madre del Sur province); TAM (Tamaulipas province); PL (Pacific Lowlands province); VE (Veracruzan province).

**Table 4:** The analysis of variance by principal component.

Groups	Differences	lwr	upr	*P* adj
**PC1**				
×*Schombolaelia*–*Laelia* s.s.	−0.8845211	−2.937996	1.168954	0.5691817
*Schomburgkia–Laelia* s.s.	−4.7513664	−5.185570	−4.317163	0.0000000 **
*Schomburgkia*–×*Schombolaelia*	−3.8668453	−5.932416	−1.801274	0.0000395 **
**PC2**				
×*Schombolaelia*–*Laelia* s.s.	−1.6353768	−3.94528089	0.6745272	0.2200067
*Schomburgkia–Laelia* s.s.	0.6413184	0.15289318	1.1297437	0.0060518 **
*Schomburgkia*–×*Schombolaelia*	2.2766952	−0.04681523	4.6002057	0.0562423

** Significance level of *p* < 0.05.

## Data Availability

Not applicable.

## Supplementary Materials

The following supporting information can be downloaded at: https://www.mdpi.com/article/10.3390/plants11202742/s1. Table S1: Bioclimatic and topographic variables for the ecological niche models of the species of the genus *Laelia*. Table S2: Bioclimatic and topographic layers used for each of the final ecological niche analyses. Table S3: Contribution of environmental variables for each PC axis and significance level in divergence in environmental variables. Figure S1: Contribution of variables to (a) component 1 and (b) component 2.
