# Supplementary material for: Patterns of Species Richness and Distribution of the Genus Laelia s.l. vs. Laelia s.s. (Laeliinae: Epidendroideae: Orchidaceae) in Mexico: Taxonomic Contribution and Conservation Implications
Source: Plants (Basel). 2022 Oct 17;11(20):2742. doi: 10.3390/plants11202742 (PMC9609499; doi:10.3390/plants11202742)
Supplement: Supplementary file 1 [file plants-11-02742-s001.zip › plants-1889848-supplementary.pdf]

plants

MDPI

Supplementary Material

Patterns of Species Richness and Distribution of the Genus *Laelia* s.l. vs *Laelia* s.s. (Laeliinae: Epidendroideae: Orchidaceae) in Mexico: Taxonomic Contribution and Conservation Implications

Ma. Isabel Olivares-Juárez, Mireya Burgos-Hernández, and Margarita Santiago-Alvarádo

Table S1. Bioclimatic and topographic variables for the ecological niche models of the species of the genus *Laelia*.

Variables	Description
bio 1	Average annual temperature
bio 2	Daytime temperature range
bio 3	Isothermality (BIO2/BIO7) (* 100)
bio 4	Seasonality in temperature (standard deviation * 100)
bio 5	Maximum temperature of the warmest month
bio 6	Minimum temperature of the coldest month
bio 7	Annual temperature range (BIO5-BIO6)
bio 8	Average temperature in the rainiest quarter
bio 9	Average temperature in the driest quarter
bio 10	Average temperature in the warmest quarter
bio 11	Average temperature in the coldest quarter
bio 12	Annual precipitation
bio 13	Precipitation in the wettest month
bio 14	Precipitation in the driest month
bio 15	Seasonality in precipitation (coefficient of variation)
bio 16	Precipitation of wettest quarter
bio 17	Precipitation of driest quarter
bio 18	Precipitation of warmest quarter
bio 19	Precipitation of coldest quarter
elevation	Elevation (m)
orientation	Orientation (%)
slope	Slope (°)
annualPET	Annual potential evapotranspiration

Table S2. Bioclimatic and topographic layers used for each of the final ecological niche analyses.

Species	Variables used
Laelia albida	bio 1, bio 5, bio 8, bio 9, bio 10, bio 14, bio 15, elevation
Laelia anceps	bio 1, bio 3, bio 4, bio 10, elevation
Laelia autumnalis	bio 1, bio 5, bio 6, bio 8, bio 9, bio 10, bio 11, elevation, PET
Laelia crawshayana	bio 1, bio 3, bio 8, bio 9, bio 10, bio 11, elevation, PET
Laelia eyermaniana	bio 1, bio 4, bio 5, bio 7, bio 8, bio 9, bio 10, bio 11, elevation, PET
Laelia furfuracea	bio 1, bio 2, bio 5, bio 7, bio 8, bio 10, bio 12, bio 17, bio 19, elevation, PET
Laelia gouldiana	bio 1, bio 4, bio 5, bio 8, bio 9, bio 10, bio 11, bio 12, bio 13, bio 14, bio 16, bio 17, bio 18, bio 19, elevation, PET
Laelia halbingeriana	bio 1, bio 8, bio 10, slope
Laelia rubescens	bio 2, bio 3, bio 4, bio 6, bio 12, bio 14, bio 15, bio 16
Laelia speciosa	bio 1, bio 3, bio 5, bio 6, bio 8, bio 9, bio 10, bio 11, bio 12, bio 18, elevation, PET
Laelia superbiens	bio 1, bio 5, bio 6, bio 8, bio 9, bio 10, bio 11, bio 18, elevation

Table S3. Contribution of environmental variables for each PC axis and significance level in divergence in environmental variables.

Variables	PC1	PC2
Elevation	0.945604310748474	0.205867239928414
Bio1	-0.925665490725553	-0.354611179948879
Bio8	-0.909198517912652	-0.378245122491003
Bio10	-0.904740628283967	-0.403069485023755
Bio12	-0.579470536860114	0.702930098046169
annualPET	-0.509764518099002	-0.693693865486644
Bio14	-0.566933273534356	0.654307021197404
Bio17	-0.571061384140902	0.664022967932186
% explained variance	38.9%	21.1%
Observed differences	**-4.643354****	0.5777236

**Significant differences in the climatic variables of *Laelia* s.s. and *Schombugkia* on each PC axis.

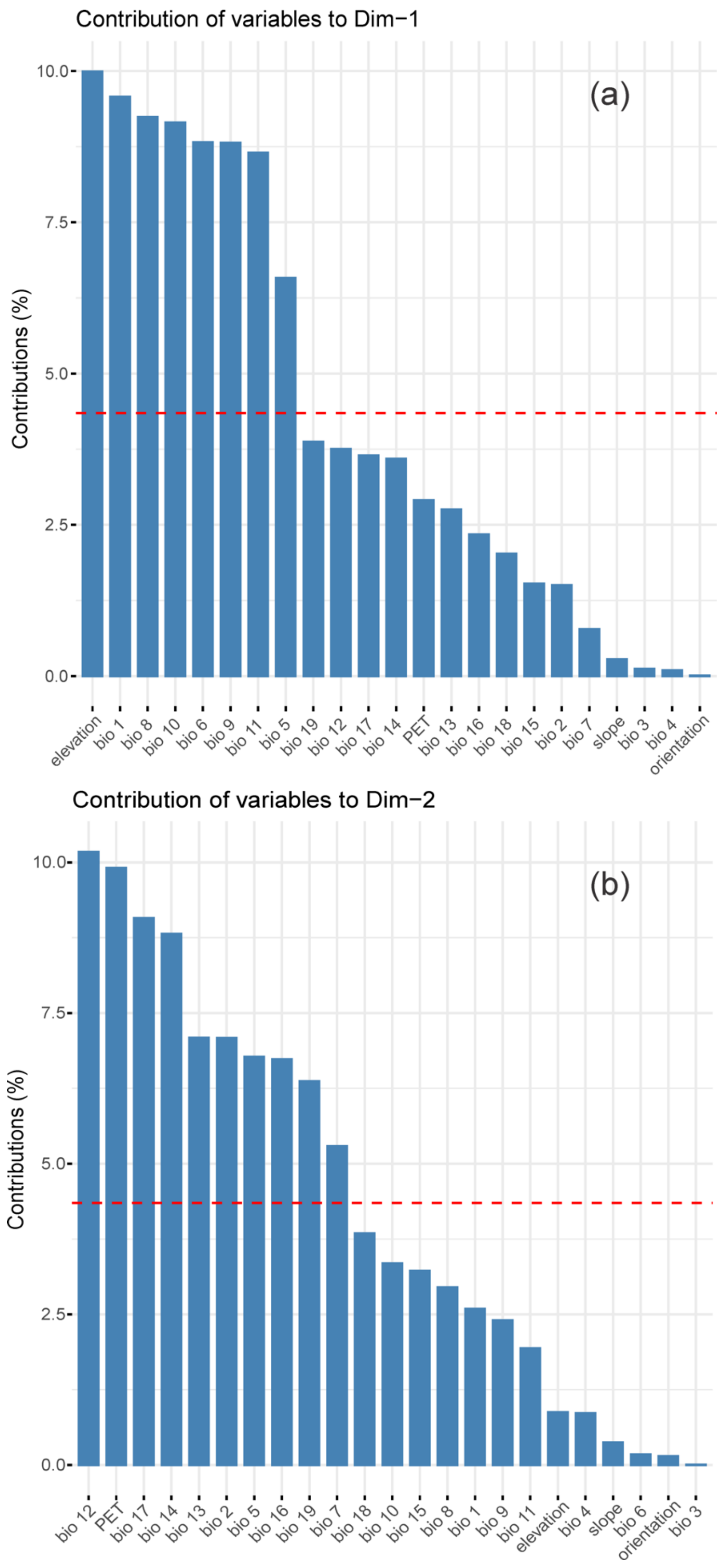

Figure S1. Contribution of variables to a) component 1, and b) component 2.
